# Globalization and Infectious Diseases in Women^1^

**DOI:** 10.3201/eid1011.040485

**Published:** 2004-11

**Authors:** Carol Bellamy

**Affiliations:** * United Nations Children's Fund, New York, New York, USA

**Keywords:** globalization, women’s health, UNICEF, HIV/AIDS, malaria, developing countries, commentary

## Abstract

Women have an enhanced vulnerability to disease, especially if they are poor. Indeed, the health hazards of being female are widely underestimated. Economic and cultural factors can limit women's access to clinics and health workers. The World Health Organization (WHO) reports that less is spent on health care for women and girls worldwide than for men and boys. As a result, women who become mothers and caretakers of children and husbands often do so at the expense of their own health. The numbers tell the story: the latest (2003) World Health Report showed that, globally, the leading causes of death among women are HIV/AIDS, malaria, complications of pregnancy and childbirth, and tuberculosis.

One might have thought that by the year 2004, gender myopia would be far less of a factor. For we now know that only by opening up educational, economic, social, and political opportunities for women can the world ensure progress in stabilizing population growth, protecting the environment, and improving human health, starting with the well-being of young children.

All these links and more were set forth in compelling terms in 1990, at the watershed World Summit for Children, where world leaders vowed to give every child a better future, and in subsequent global gatherings on environment, population, and the pervasive challenges to human rights, including the rights of women and girls. More recently, those commitments were reaffirmed by governments in connection with their embrace of the United Nations (U.N.) Millennium Development Goals, the action plan of the 2002 General Assembly Special Session on Children, and United Nations Children's Fund (UNICEF) Medium Term Strategic Plan.

Moreover, the positive aspects of globalization have begun to make a difference in areas where women suffer disproportionately, especially from two leading diseases with high death rates, malaria, and HIV/AIDS. Malaria, for example, can be prevented and treated by available cost-effective interventions, including insecticide-treated nets that can cut malaria deaths by 20% and reduce infections by 50%. During pregnancy, malaria complications and deaths can be prevented by administering two doses of an antimalarial drug (sulfadoxine-pyrimethamine) during the first and second trimesters.

UNICEF is working with WHO, the World Bank, the Global Fund for AIDS, Tuberculosis and Malaria, and other Roll Back Malaria partners to support malaria-endemic countries to ensure increased use of insecticide-treated nets, access to effective antimalarial drugs and treatment, and prevention and control of malaria epidemics. During 2003, UNICEF procured >5 million insecticide-treated nets for 25 countries in Africa. Most of the nets were distributed to pregnant women, through antenatal clinics, and to children <5 years of age during routine childhood immunization and measles vaccination campaigns.

Meanwhile, the struggle to curb the spread of HIV/AIDS has benefited from a gradual rise in resources coincident with the effort of WHO, UNICEF, and other partners to place 3 million patients in sub-Saharan Africa into treatment programs by 2005. Moreover, the price of generic antiretroviral drugs has fallen sufficiently for African governments, backed by external resources, to begin prolonging the lives of their citizens.

But in many of the countries of eastern, central, and southern Africa, the AIDS pandemic has already reversed many of the development gains of the last decade—so much so that the Millennium Development Goals the United Nations has targeted for 2015 are already moot. Life expectancy in many countries has dropped from an average 60–62 to age 37–40. Infant death rates in the region are up; children are leaving school to care for sick and dying parents, while whole sectors of society—agriculture, health, education, the private sector—are diminished and compromised by the loss of their most productive workers in what should be the prime of life.

As Stephen Lewis, Special Envoy of the Secretary-General for AIDS in Africa, said recently, "It is an astonishing tribute to the people of Africa—their resilience and their determination—that countries continue to function, heroically, even as they are assaulted by the pandemic." This situation is occurring against a backdrop of immense economic and social inequity. The world economy is worth >$30 trillion. Yet nearly half of humanity, 2.8 billion people, most of them women and children, live on <$2 a day.

This almost unimaginable impoverishment and lack of employment make the idea of health and health care for women even less attainable. The situation is made worse by high female illiteracy rates in many countries and increasing cuts in government aid for life-saving malaria, HIV/AIDS, and tuberculosis drugs. These factors have led to rampant spread of infectious diseases in the world's poorest communities.

HIV/AIDS is still holding firm as the worst communicable disease in history. The virus is now the leading cause of death in Africa and the fourth leading cause of death worldwide. Africa south of the Sahara has been the worst affected region. Some countries in Asia and eastern Europe are also showing rapid increases in HIV/AIDS prevalence. The fastest rate of infection is among teenage girls. Older men usually infect younger women, either through early marriages for girls or through prostitution.

Any understanding of the gender-based aspects of HIV infection must take into account issues of power, human rights, and social and cultural expectations. A U.N. expert group that met to discuss gender implications of the pandemic concluded that the rapid spread of HIV infection and its deleterious effects on families, communities, and countries were a direct outgrowth of women's inequality and lack of power at all levels.

On the other hand, we know that the active involvement of women and girls in their own well-being is key to bringing about more effective prevention and control of HIV/AIDS. The lack of control by women and girls over their bodies and sex lives, in the context of general socioeconomic subordination, places women all over the world in a more vulnerable situation in relation to HIV/AIDS.

Violence against women is a major risk factor for HIV/AIDS. Infection rates are usually linked to the incidence of rape and other unprotected sexual incidents, especially in times of armed conflict and civil unrest. Women often do not have the power to refuse unwanted sex or to negotiate safe sex with their partners because of fear of violence.

The high death rate among women from HIV/AIDS can be devastating in many countries because of the role women play in child and family survival and community development. Loss of a mother in many parts of the developing world usually means that her young children will die as well, especially those <5 years of age. Orphaned children are most likely to be poorly nourished, miss school, experience emotional trauma, and make themselves more vulnerable to the virus.

Because of this disease, and the social stigma and resulting silence surrounding it, women and children are suffering and dying in ways and in numbers that no earlier generation could have imagined possible. Yet we are confronting a disease that is 100% preventable.

UNICEF has three overarching goals. The first is reducing HIV infection among young people. Our absolute, immutable priority remains the prevention of this disease. And so, in collaboration with young people, U.N. sister agencies, and other partners, UNICEF wants to ensure that every young person has access to basic information on how to avoid infection and that programs are in place to make this information available by 2005. This plan includes access to confidential testing, counseling, and youth-friendly health services that can offer frank information about how sexually active young people can protect themselves and their partners.

Second, we are committed to expanding care and support for orphans and other children made vulnerable by HIV. We need to scale-up alternative forms of care so that such children do not grow up alone, but in familylike environments, with protection, love, and care.

Third, we must reduce mother-to-child transmission. This multifaceted process includes elements of both prevention and treatment. The first step is to prevent infection in women of childbearing age. In some countries, adolescent girls are six times more likely than boys to get infected. This situation is a direct consequence of gender inequality and sexual abuse.

We need to provide women with voluntary and confidential counseling and testing. If they are HIV-positive, they must be given access to antiretroviral drugs to reduce viral loads and chances of infecting their infants. At the same time, they need counseling and advice on feeding options.

Reducing mother-to-child transmission also offers us a foothold in tackling broader treatment, which is both possible and an essential part of the campaign against HIV/AIDS. Success of the immunization campaign, spearheaded by UNICEF and WHO, shows what can be done with antiretroviral drugs in terms of funding, procurement, and distribution.

For now, UNICEF remains convinced that, until an effective medical remedy is found, education is the only effective tool for curbing HIV/AIDS. Only education can empower young people with the knowledge they need to protect themselves and their communities. Only education can combat the discrimination that helps perpetuate the pandemic. And only education can help children and young people acquire the knowledge and develop the skills they need to build a better future, the better future that the international community promised every child a decade ago, at the World Summit for Children. UNICEF is working with governments in more than 161 countries to promote the welfare of children and women. The Millennium Development Goals, set by the United Nations in 2000, call for reducing the under-5 childhood death rate by two thirds from 1990 to 2015; reducing maternal deaths by three quarters from 1990 to 2015; and reversing the spread and incidence rates of HIV/AIDS, malaria, and other infectious diseases by 2015. Controlling major infectious diseases such as malaria, HIV/AIDS, and others among women and children will be crucial to attaining the key Millennium Development Goals. It is crucial that we work to find ways to improve the socioeconomic status of women, beginning with ensuring their right to education, especially for girls. This effort must also include providing access to clean water and adequate sanitation, health facilities and life-saving drugs, and land and credit as well as promoting women's right to active involvement in the affairs of their community.

Globalization can have a positive impact on children and their families, and its negative effects can be minimized. The challenge is how to bring those benefits, such as new health technologies, to vulnerable groups, especially children, women, and marginalized populations, to prevent and control major infectious diseases such as malaria and HIV/AIDS. The HIV/AIDS pandemic has a woman's face, and if women and girls are not empowered, especially in terms of their own sexuality, the pandemic will never end.

